# Dihydromyricetin protects against cisplatin-induced renal injury and mitochondria-mediated apoptosis via the EGFR/HSP27/STAT3 signaling pathway

**DOI:** 10.1080/0886022X.2025.2490202

**Published:** 2025-04-14

**Authors:** Zheming Xu, Minjing Zhang, Xue Zhang, Huirong Han, Weifeng Ye, Zhenjie Chen, Zhisu Lv, Yang Liu, Zhengye Liu, Jianguang Gong, Bin Zhu, Suhan Zhou, Runzhi Zhu, Chang Tao, Gensheng Zhang, Xiang Yan

**Affiliations:** ^a^ Department of Urology, Pediatric Urolith Center, Children’s Hospital, Zhejiang University School of Medicine, National Clinical Research Center for Child Health, Hangzhou, China; ^b^ Children’s Hospital, Zhejiang University School of Medicine, National Clinical Research Center for Child Health, National Children’s Regional Medical Center, Hangzhou, China; ^c^ School of Anesthesiology, Shandong Second Medical University, Laboratory of Anesthesia and Critical Care Medicine in Colleges and Universities of Shandong Province, Weifang, China; ^d^ Department of Plastic and Aesthetic Center, The First Affiliated Hospital, College of Medicine, Zhejiang University, Hangzhou, China; ^e^ Urology & Nephrology Center, Department of Nephrology, Zhejiang Provincial People’s Hospital, Affiliated People’s Hospital, Hangzhou Medical College, Hangzhou, China; ^f^ Department of Physiology, School of Basic Medical Sciences, and Kidney Disease Center of the First Affiliated Hospital, Zhejiang University School of Medicine, Hangzhou, China

**Keywords:** Cisplatin, acute kidney injury, dihydromyricetin, oxidative stress, HSP27

## Abstract

**Background:**

Cisplatin (CP) has been used as an effective chemotherapy drug for different types of cancers. Despite its therapeutic benefits, the clinical utility of CP is often hindered by adverse effects, notably acute kidney injury (AKI), which restricts its widespread application. Dihydromyricetin (DHM) is a flavonoid acquired from *Ampelopsis grossedentata*, exhibiting a range of pharmacological activities. The major objective of this research was to examine the possible molecular mechanism involved in CP-induced AKI and the protective function of DHM.

**Methods:**

In this study, the protective function of DHM against CP-induced AKI was assessed in both mice and HK-2 cells. Kidney dysfunction parameters and renal morphology were evaluated to ascertain the extent of protection. Additionally, proteomics techniques were employed to investigate the protective effect of DHM and elucidate the underlying molecular mechanisms involved in mitigating CP-induced AKI. In addition, protein levels of epidermal growth factor receptor (EGFR), p-EGFR, heat shock protein 27 (HSP27), p-HSP27, STAT3, and p-STAT3 in renal tissues were investigated. Furthermore, an EGFR-blocking agent (gefitinib) or si-RNA of HSP27 was used to study the effects of inhibiting EGFR or HSP27 on CP-induced renal injury.

**Results:**

DHM decreased blood urea nitrogen (BUN) and creatinine in serum, alleviated renal morphological injury and downregulated the expression of CP-induced kidney injury molecule-1 and neutrophil gelatinase-related lipocalin. Proteomic data revealed HSP27 as a potential therapeutic target for AKI. DHM treatment resulted in the downregulation of EGFR, HSP27, and STAT3 phosphorylation, ultimately mitigating CP-induced AKI. In addition, the inhibition of EGFR or HSP27 reduced mitochondria-mediated apoptosis and CP-induced cell damage in HK-2 cells.

**Conclusions:**

DHM effectively inhibited CP-induced oxidative stress, inflammation, and mitochondria-mediated apoptosis through the EGFR/HSP27/STAT3 pathway.

## 1. Introduction

Cisplatin (CP) is a widely used first-line anti-cancer drug to treat malignant tumors including the head and neck, ovaries, testis, bladder, cervix, lung carcinoma, and other types of cancer [1–3]. However, one of the primary adverse effects of CP is nephrotoxicity, primarily resulting from its accumulation in proximal tubular epithelial cells [4]. About 30% of individuals who are treated with CP experience acute kidney injury (AKI) [5–7]. Reactive oxygen species (ROS), inflammation, oxidative stress, apoptosis, necrosis, and mitochondrial dysfunction in the tubular epithelial cells contribute to this process [6,8–12]. Despite several studies on understanding the mechanisms of CP-induced AKI, current clinical strategies for its prevention or treatment are limited. Thus, developing potential therapeutic drugs for preventing CP-induced AKI is urgently required.

The epidermal growth factor receptor (EGFR) belonging to the ErbB receptor tyrosine kinase family, is expressed throughout the mammalian kidney [13]. It has intrinsic tyrosine kinase activity [14] due to which it can undergo self-phosphorylation following receptor dimerization. The phosphorylation of EGFR triggers a series of intracellular events, including phosphatidylinositol 3-kinase/protein kinase B (PI3K/PKB) [15,16], mitogen-activated protein kinase (MAPK) [17,18], and heat shock protein 27 (HSP27) [19]. Increasing evidence indicates that the activation of EGFR contributes to different types of AKI [20–23]. HSP27 emerges as a prominent heat shock protein showing increased expression in renal dysfunction [24–27]. It has been implicated in renal-associated pathological processes such as oxidative stress [28], inflammation [29], autophagy [30], and apoptosis [31,32]. Signal transducer and activator of transcription 3 (STAT3), downstream signaling of EGFR and HSP27, has been reported to be associated with AKI [33,34] or chronic kidney disease (CKD) [35]. Blockage of EGFR or its downstream signaling pathways, such as HSP27 and STAT3, can have therapeutic potential for inhibiting the progression of renal injury.

Dihydromyricetin (DHM) is a flavonoid found in several plants and is an active ingredient in several traditional Japanese, Chinese, and Korean medicines [36]. DHM has been demonstrated to exert a protective effect in certain AKI models [37–39]. However, research on the specific molecular mechanism of DHM in CP-induced AKI is limited [40,41]. Our previous study showed that DHM exhibited protective effects through alleviating oxidative/nitrative stress, inflammation, apoptosis, and ferroptosis probably by targeting classic signaling pathways such as Nrf2/HO-1, MAPK and NF-κB. This study aims to explore a novel mechanism of DHM in improving CP-induced AKI and new targets for AKI treatment through proteomic techniques.

## 2. Materials and methods

### 2.1. Chemicals and antibodies

DHM (Meica-0826) was obtained from Nanjing Meica Bio-Pharm Technology (Nanjing, China), and CP (HY-17394) was bought from MedChemExpress (New Jersey, USA). CP was added to 0.9% saline for all tests while DHM was added to dimethyl sulfoxide (DMSO) or 0.9% saline for cell or animal experiments. 5-(and-6)-carboxy-2′,7′-dichlorodihydrofluorescein diacetate (H_2_DCFH-DA, #BL714A) was provided by Biosharp (Beijing, China). Anti-STAT3 (#9139 T), anti-phospho-STAT3 (#9145 T), anti-c-PARP (#5625s), and anti-Bcl-2 (#3498 T) antibodies were provided by Cell Signaling Technology (Danvers, USA). Anti-Bax (#A0207), anti-HSP27 (#A11156), anti-phospho-HSP27-s82 (#AP1031), and anti-phospho-HSP27-s78 (#AP1032) antibodies were sourced from ABclonal Technology (Wuhan, China). Anti-β-actin (#66009-1-Ig) antibody was sourced from Proteintech Group (Wuhan, China). Anti-EGFR (#sc-373746) and anti-p-EGFR (#sc-57545) antibodies were brought from Santa Cruz Biotechnology (Dallas, USA). Secondary anti-mouse IgG (#A0216) and anti-rabbit IgG (#A0208) antibodies were sourced from Beyotime Biotechnology (Shanghai, China).

### 2.2. Animal models of CP-induced AKI

Male C57BL/6 mice (10 weeks) from *GemPharmatech* (Nanjing, China) were kept in standard cages under controlled temperature (22 °C–24 °C) in a 12-h light/12-h dark cycle. They were provided *ad libitum* access to water and food. After approximately one week of acclimatization, mice were stratified randomly into sham, CP, and CP + DHM categories. Mice were treated with DHM (500 mg/kg) or 0.9% saline by gavage for about 7 d before CP treatment and continued until mice termination. A single dose of CP (22 mg/kg) [41,42] was intraperitoneally injected to induce a murine AKI model. The mice were euthanized at 72 h after CP injection. The Animal Ethics Committee of Zhejiang University School of Medicine approved all the experiments performed in this research.

### 2.3. Blood urea nitrogen (BUN) and creatinine detection

Mice were administered anesthesia utilizing isoflurane. Blood samples were collected *via* the inferior vena cava. All samples underwent centrifugation as previously described [43,44] and supernatants were preserved immediately at −80 °C. Serum creatinine and BUN were examined in the samples using an automatic biochemical analyzer (Roche, Switzerland) at the Center for Drug Safety Evaluation and Research of Zhejiang University College of Pharmaceutical Sciences.

### 2.4. Histopathological assessment

First, 4% paraformaldehyde was utilized for kidney tissue fixation, and the tissues were subsequently embedded in paraffin. The renal sections were sectioned at 4 μm intervals and stained employing the kits purchased from Servicebio (Wuhan, China) for periodic acid-schiff (PAS) staining. Tubular damage was assessed based on the following parameters: tubular brush border loss, tubular dilatation and disruption, flattened epithelial cells, and sloughing of tubular epithelial cells. The quantification of tubular damage was evaluated in a blinded manner and scored based on the percentage of injured tubules: no damage (0), mild (1, <25%), moderate (2, 25%–50%), severe (3, 50%–75%), and very severe injury (4, >75%) [45,46]. Ten fields were randomly chosen from each section to assess the extent of the injury.

### 2.5. Transmission electron microscopy (TEM)

Electron microscopy samples were processed and examined at the Center of Cryo-Electron Microscopy (CCEM), Zhejiang University School of Medicine. The samples were sectioned, stained, and observed under an electron microscope as previously described [45,47]. In brief, kidney tissues were sectioned into small pieces and promptly fixed with 2.5% glutaraldehyde at 4 °C. The specimens underwent triple rinsing with 0.1 M phosphate-buffered saline (PBS) and were subsequently fixed with 1% osmic acid for 1 h and 2% uranium acetate for 0.5 h. Subsequently, the samples underwent dehydration with a series of ethanol concentrations (50%, 70%, 90%, and 100%) and 100% acetone, before being embedded in epoxy resin along with hardener.

### 2.6. Immunofluorescence staining

The kidneys were processed using Tissue-Tek O.C.T (SAKURA, Japan) and frozen sections (5 μm) were then prepared and further processed. The sample slices underwent washing with 1 × PBS and were subsequently blocked with blocking buffer (5% bovine serum albumin [BSA] in 1 × TBST) for 30 min as described earlier [48]. The samples were then incubated at 4 °C overnight with the prepared primary antibodies including anti-KIM-1 (#AF1817, R&D), anti-Lipocalin-2/NGAL (#A2092, ABclonal), anti-IL1Ra (#A11103, ABclonal), BPIFA2 (#NBP2-37493, NOVUS), anti-HSP27 (#A11156, Abclonal), anti-p-STAT3 (#9145 T, CST), anti-TNF-α (60291-1-Ig, Proteintech) and anti-IL-1β (#A1112, Abclonal). The samples were next incubated with secondary antibodies (ABclonal, AS032, 1:200 for KIM-1; Beyotime, A0423, 1:500 for NGAL, IL-1Ra, HSP27, and p-STAT3; Proteintech, SA00013-3, 1:200 for BPIFA2 and TNF-α; ABclonal, AS039, 1:200 for IL-1β) at room temperature for 2 h. Followed by washing with 1 × TBST three times and then briefly with water. Afterward, the samples were mounted with an antifade mounting medium for fluorescence with DAPI (Biosharp, China). Tissues were imaged utilizing a laser confocal microscope (TCS SP8, Leica, Germany). The immunofluorescence intensity was measured utilizing ImageJ (NIH, USA).

### 2.7. Immunoblotting

Proteins were isolated from renal tissues or HK-2 cells with cell lysis buffer for western and immunoprecipitation (P0013J, Beyotime Biotechnology, Shanghai, China) with phosphatase and protease inhibitors. The lysates were centrifugated at 4 °C for 10 min with 12,000 × g. Protein concentration was quantified utilizing the BCA protein assay kit (MA0082, MeilunBio, Dalian, China). An equal quantity of proteins was loaded in each lane for sodium dodecyl sulfate-polyacrylamide gel electrophoresis (SDS-PAGE) and immunoblot analysis as elaborated previously [44,49]. The GeneSys Chemi system (Syn-gene G: BOX, USA) was used to visualize the membranes. The band intensity was measured utilizing Image J (NIH, USA).

### 2.8. Proteomics

Briefly, proteins were extracted from kidney samples taken from mice using RIPA lysis buffer with sonication at 4 °C and quantified using the BCA assay bought from Beyotime Biotechnology (Shanghai, China) following the manufacturer’s instructions. Samples were mixed with ice acetone and incubated at −20 °C overnight. The samples were then centrifuged at 13,000 rpm at 4 °C for 10 min and subsequently washed with 80% acetone. Proteins were enzymatically digested into peptides, and a freeze dryer was used to convert the peptide solution into a powder. According to the labeling reagent protocol of the TMT label kit (Thermo Fisher Scientific), equal amounts of proteins were labeled with TMT according to the protocol. After desalting, the labeled peptide segment powder was dissolved. Lastly, TMT-Labeled Mass Spectrometry Analysis of renal samples was performed by Shanghai BIOTREE BIOTECH Co. Ltd (Shanghai, China).

### 2.9. Cell culture

HK-2 cells, obtained from ATCC, were cultured in Dulbecco’s modified Eagle’s medium (DMEM)/F12 medium with 10% fetal bovine serum (BSA) and 1% streptomycin and penicillin mixture in an incubator with 5% CO_2_ at 37 °C. The cells were plated in a 10 cm dish or 6-well plate overnight and then treated with DHM or vehicle (DMSO) for 24 h and subjected to CP to mimic CP-induced AKI. The concentrations of DHM and CP used in the experiment are 100 μM and 30 μM, respectively according to our previous study [41]. Lastly, the cells were collected for further analysis.

### 2.10. EGFR or HSP27 inhibition experiment

The cells were treated with DMSO, gefitinib (Gef, an EGFR inhibitor, HY-50895, MedChemExpress, USA), si-NC, or si-HSP27 (GenePharma, China) for EGFR or HSP27 inhibition over a 24-h period. Subsequently, the cells were treated with CP for a further 24 h. Finally, the cell samples were collected for western blotting or flow cytometry. Flow cytometry was utilized to detect the percentage of annexin V-positive apoptotic cells (BD FACSLyric Flow Cytometer, USA) after staining the cells with an Apoptosis Detection Kit (#556547, BD Pharmingen, USA).

### 2.11. Intracellular ROS assessment

A fluorescent probe, H_2_DCFH-DA, was utilized to assess the intracellular levels of ROS in HK-2 cells. HK-2 cells were incubated with H_2_DCFH-DA (10 µM) at 37 °C for 30 min modified according previously described [50]. The ROS levels were determined by taking fluorescence images using a confocal microscope. The immunofluorescence intensity was measured utilizing Image J (NIH, USA).

### 2.12. Statistical analysis

The acquired data are expressed as the mean ± standard error of the mean (SEM). Comparative analysis among various categories was made utilizing a one-way analysis of variance (ANOVA), followed by Tukey’s multiple-comparison test. *p* < .05 was determined to denote statistical significance. GraphPad Prism was used for certain analyses.

## 3. Results

### 3.1. DHM ameliorates renal injury following CP-induced AKI in mice

**Figure 1.**
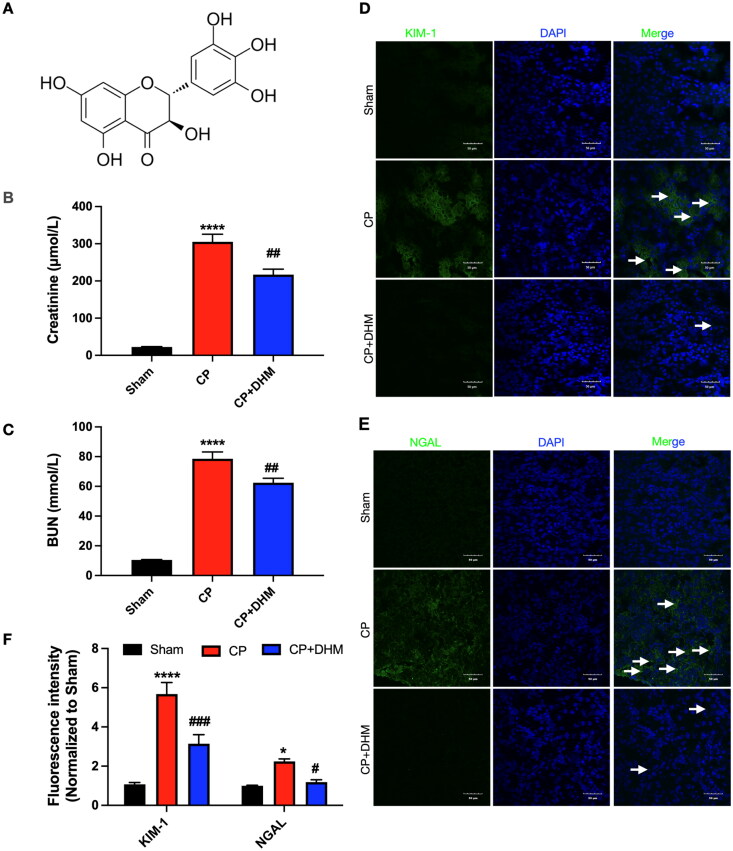
Effect of DHM on renal injury after CP-induced AKI *in vivo.* (A) molecular structure of DHM. (B and C) Creatinine and BUN levels in serum after cisplatin (CP) injection (*n* = 6/group). (D and E) KIM-1 and NGAL levels were detected using immunofluorescence staining (scale bar, 50 μm, *n* = 3/group). (F) Evaluation of normalized KIM-1 and NGAL intensities. The one-way ANOVA method was used to analyze the significance difference of multiple groups. *, ****p < .05 and .0001, respectively *versus* the sham group; #, ##, ###*p* < .05, .01, and .001, respectively *versus* the CP group.

Initially, the protective role of DHM in renal injury was investigated through a CP-induced AKI mouse model. CP significantly increased renal dysfunction markers, including serum creatinine and BUN, which were prevented by DHM (Figure 1(B,C)). Moreover, immunofluorescence results demonstrated elevated protein expression of kidney injury molecule-1 (KIM-1) and neutrophil gelatinase-associated lipocalin (NGAL), two markers of renal proximal tubular injury, in comparison to the sham category, while DHM considerably decreased their levels (Figure 1(D–F)).

### 3.2. Effects of DHM on renal morphology after CP-induced AKI in mice

**Figure 2.**
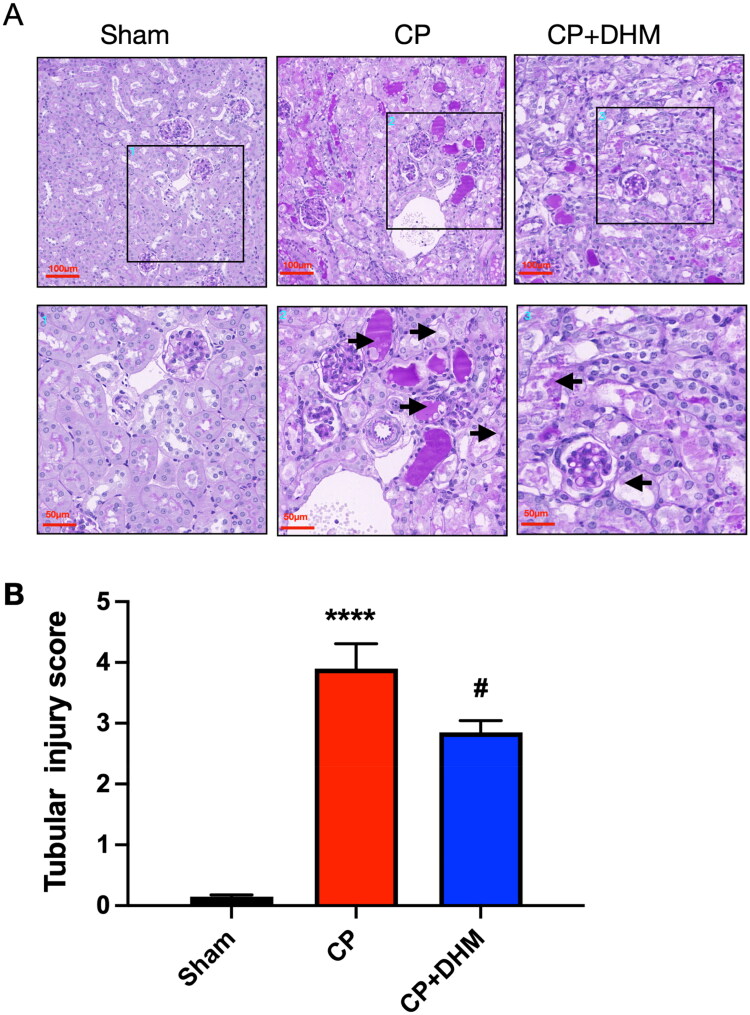
Effect of DHM on renal histopathology after CP-induced AKI *in vivo.* (A) Representative images of PAS staining (scale bar, 100 μm, and 50 μm, *n* = 3). (B) Tubule cell injuries were scored from 0 to 4. ****p < .0001 *versus* the sham group; #*p* < .05 *versus* the CP group.

Next, the renal histopathological alterations were analyzed after AKI using PAS staining. The data implied that the CP group displayed a greater degree of morphological damage, such as tubular brush border loss, tubular dilation and disruption, cast formation, and swelling and necrosis compared with the sham group. These changes were markedly reduced in AKI mice pretreated with DHM (Figure 2(A,B)).

### 3.3. DHM alleviates renal mitochondrial damage, mitochondrial-mediated apoptosis and the inflammatory response in CP-induced AKI in mice

**Figure 3.**
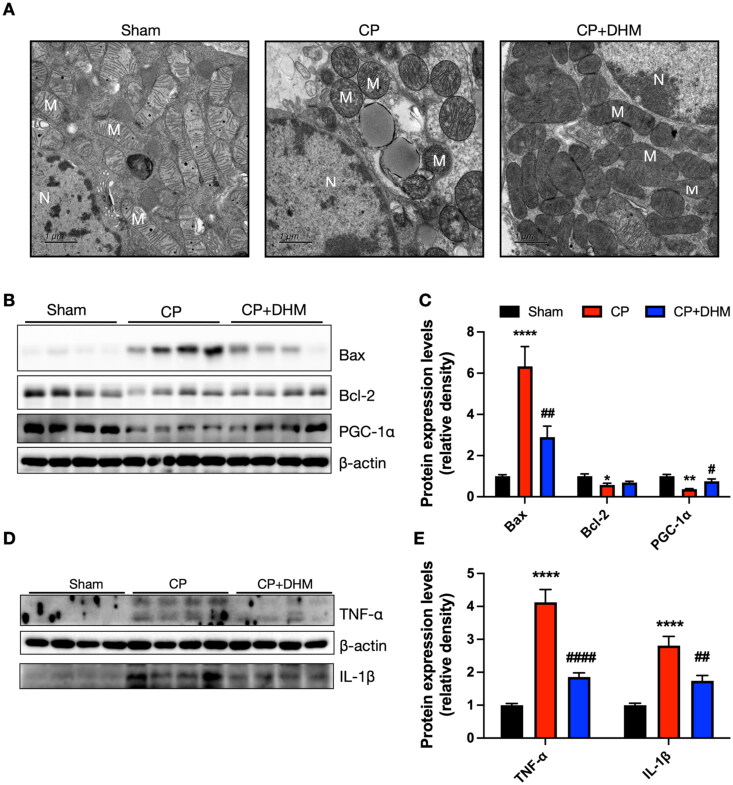
Effect of DHM on CP-induced renal inflammation and mitochondrial dysfunction/injury *in vivo*. (A) Representative TEM images in renal tissues in three categories (M: mitochondria; N: nucleus. Scale bar, 1 μm, *n* = 3/category). TEM showed swollen mitochondria and ruptured mitochondrial cristae in CP-treated mice, which were obviously attenuated by DHM. (B) Representative immunoblotting images of bax, bcl-2, and PGC-1α. (C) Evaluation of normalized bax, bcl-2 and PGC-1α (*n* = 6/category). (D) Immunoblotting images of TNF-α and IL-1β. (E) Evaluation of normalized TNF-α and IL-1β (*n* = 6/category). *, **, ****p < .05, .01, .0001, respectively *versus* sham category; #, ##, ####*p* < .05, .01 and .0001, respectively *versus* CP category.

During kidney tubular cell injury, mitochondria dysfunction causes cell apoptosis. Because the mitochondrion is the primary target of CP-induced AKI [51,52], the mitochondrial structure change in tubular epithelial cells was further examined by TEM analysis. The TEM data showed that mitochondria exhibited intact cristae morphology in the normal group, whereas those in the CP-AKI category were damaged and characterized by shortening, swelling, and globular morphology. DHM largely alleviated mitochondrial morphological changes induced by CP treatment (Figure 3(A)).

It has been indicated that heightened expression of peroxisome proliferator-activated receptor gamma coactivator 1-alpha (PGC-1α) increases mitochondrial number and alleviates kidney injury [53,54]. Recent investigations have implied that PGC-1α is crucial in CP-induced AKI [55,56]. Mitochondrial apoptosis was observed to be a prominent feature in CP-induced AKI [57,58]. In this investigation, mitochondrial apoptosis-related protein levels in renal tissues were compared between the three categories. CP elevated the pro-apoptotic protein Bax while decreasing the anti-apoptotic protein Bcl-2. DHM treatment prevented the activation of Bax (Figure 3(B,C)). In addition, CP inhibited the protein level of PGC-1α in mice, while DHM significantly upregulated its expression (Figure 3(B,C)). Together, these data suggest that DHM alleviated CP-induced mitochondrial dysfunction and mitochondrial-mediated apoptosis.

Tumor necrosis factor (TNF)-α and IL-1β are members of inflammatory responses. Our immunoblot data demonstrated that TNF-α and IL-1β were considerably increased in CP-treated mice in comparison to the sham category, which were restored partially in AKI mice treated with DHM (Figure 3(D,E)). Consistent with the Western blot results, our immunofluorescence data showed that TNF-α and IL-1β were remarkably increased after treatment with cisplatin compared with the sham group, which was inhibited by DHM treatment (Figure S1A–D).

### 3.4. TMT-based proteomics data demonstrated the potential mechanism of DHM on CP-induced AKI in mice

**Figure 4.**
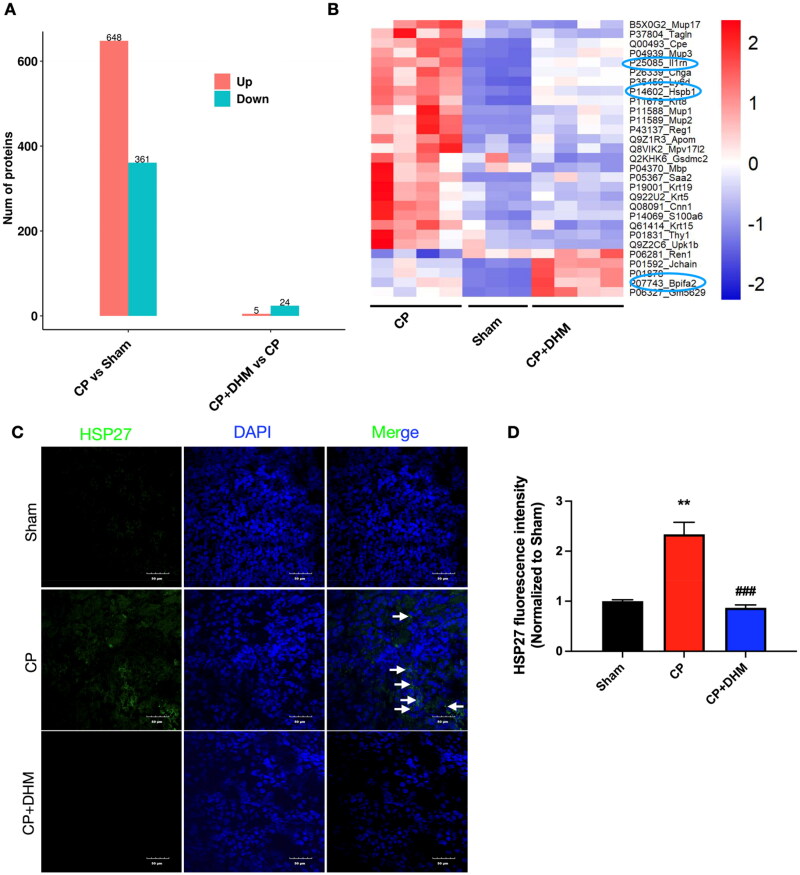
The mechanism of DHM on CP-induced AKI *in vivo* based on TMT-quantitative proteomics. (A) Histogram of statistical analysis of DEPs. (B) Heatmap representation of DEPs among the three categories. The right-side color scale corresponds to the relative protein expression (red, higher than 0; blue, less than 0) (*n* = 3–4/group). (C and D) Immunofluorescence confocal images of HSP27 (C), with quantitation displayed in (D) (*n* = 3/group). **p < .01 *versus* sham category; ###*p* < .001 *versus* CP category.

The potential mechanism of DHM against AKI was further demonstrated, for which a TMT-based proteomics assessment was conducted. The histogram of statistical analysis of differentially expressed proteins (DEPs) among the three categories depicted that: (1) compared to the sham category, 1009 DEPs were detected in the CP category (648 proteins upregulated and 361 downregulated), (2) in comparison to the CP category, 29 DEPs were detected in the Cis + DHM category (5 proteins upregulated and 24 downregulated) (Figure 4(A)). Volcano analysis revealed distinct protein expression profiles among the three categories (Figure S2A). KEGG and GO analysis data are displayed in Figure S2(B,C), respectively. Hierarchical clustering analysis is depicted as heatmaps in Figure 4(B) and Figure S2(D).

The DEPs of the three categories were compared, following which three proteins of interest were selected for further validation. Immunofluorescence staining data displayed that the expression of HSP27 (gene name: *hspb1*) was significantly increased after CP treatment, while DHM prevented the changes (Figure 4(C,D)). Moreover, IL-1Ra (gene name: *il1rn*) and BPIFA2 protein level was significantly increased after the administration of CP. DHM prevented the change of IL-1Ra, while further enhanced BPIFA2 levels (Figure S3(A–D)). All the experimental results are consistent with our proteomic data.

### 3.5. DHM attenuates CP-induced nephrotoxicity by regulating the EGFR/HSP27/STAT3 pathway in mice

**Figure 5.**
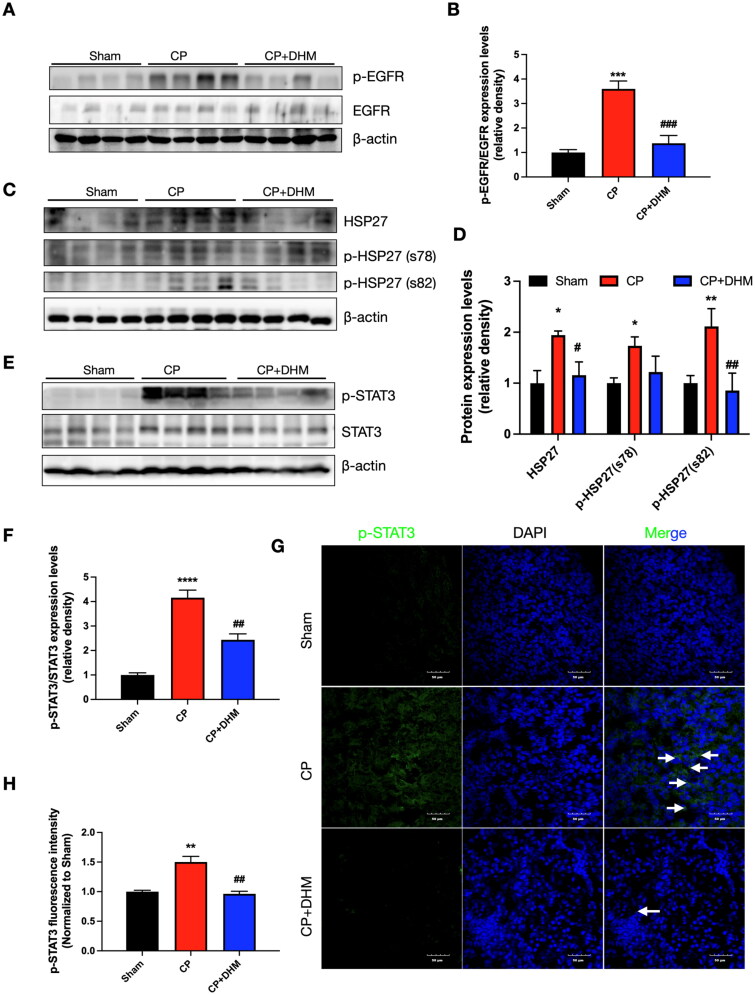
Effect of DHM on the EGFR/HSP27/STAT3 pathway after CP-induced AKI in mice. (A) Representative images of p-EGFR and EGFR in kidney tissues in three categories. (B) Evaluation of normalized p-EGFR/EGFR. (C) Representative immunoblotting images of HSP27 and p-HSP27 (s78, s82). (D) Evaluation of normalized HSP27 and p-HSP27. (E) Immunoblotting images of p-STAT3 and STAT3 protein levels in renal tissues following CP treatment. (F) Evaluation of normalized p-STAT3/STAT3. (G) Immunofluorescence images of p-STAT3. (H) evaluation of normalized p-STAT3 intensity. β-actin was utilized as the loading control. *, **, ***, ****p < .05, .01, .001, and .0001 *versus* the sham category; #, ##, ##, ####*p* < .05, .01, .001, and .0001 *versus* the CP category.

HSP27 is a member of heat shock proteins that plays multiple roles in the pathogenesis of several kidney diseases [27,29, 30,59,60]. Subsequently, the mechanisms underlying HSP27-related signaling pathways were investigated in the subsequent experiments. To delve deeper into the underlying mechanisms of DHM against CP-induced renal injury, the protein levels of EGFR, HSP27, and STAT3 were assessed in renal specimens by Western blot. EGFR activation, evidenced by increased phosphorylation following CP treatment, was reversed upon DHM administration (Figure 5(A,B)). In comparison to the sham category, CP elevated both HSP27 and p-HSP27 protein levels, whereas DHM prevented these changes (Figure 5(C,D)). A recent study demonstrated that JAK2/STAT3 functions downstream of the HSP27 pathway in liver fibrosis [61]. Therefore, the changes in p-STAT3/STAT3 protein levels were detected. Immunoblotting data showed that the ratio of p-STAT3 and STAT3 was considerably elevated, which were prevented by DHM treatment (Figure 5(E,F)). Consistent with the immunoblotting data, immunofluorescence results indicated that p-STAT3 was significantly upregulated, which was prevented following DHM treatment (Figure 5(G,H)).

### 3.6. DHM attenuates CP-induced oxidative stress, apoptosis and phosphorylation of HSP27 and STAT3 in HK-2 cells

We further demonstrated the molecular mechanism by which DHM improved renal injury *in vitro* by using HK-2 cells. Firstly, we investigated the effect of DHM on cisplatin induced oxidative stress and apoptosis. Compared to the sham category, a higher generation of ROS was noted in the kidney sections in CP-treated cells, as demonstrated by staining with H2DCFH-DA, an indicator of oxidation, whereas the ROS level significantly decreased after DHM treatment (Figure 6(A,B)). CP induced significant up-regulation of pro-apoptotic proteins Bax, and c-PARP and downregulation of anti-apoptotic protein Bcl-2, which were improved by DHM treatment (Figure 6(C,D)). Next, we detected the related proteins of the HSP27/STAT3 pathway, and the results showed that HSP27, p-HSP27 (phosphorylation sites at s78 and s82), and p-STAT3 significantly increased after CP treatment, which were prevented by DHM (Figure 6(E,F)).

Figure 6.Effect of DHM on the oxidative stress, apoptosis, and HSP27/STAT3 pathway after CP-induced HK-2 cell injury *in vitro*. (A) ROS levels were detected using H_2_DCFH-DA staining (scale bar, 100 μm, *n* = 3/category). (B) Evaluation of normalized H_2_DCFH-DA intensity. (C) Representative images of bax, bcl-2, and c-PARP protein levels in HK-2 cells. (D) Evaluation of normalized bax, bcl-2, and c-PARP (*n* = 4/category). (E) Representative images of HSP27, p-HSP27 (s78, s82), p-STAT3 and STAT3 protein levels in HK-2 cells. (F) Evaluation of normalized HSP27, p-HSP27 (s78, s82), p-STAT3 and STAT3 by using image J software (*n* = 4/category). *, **, ***, ****p < .05, .01, .001, .0001 *versus* sham category, respectively; #, ###, ####*p* < .05, .001, .0001 *versus* CP category, respectively.

**Figure:**
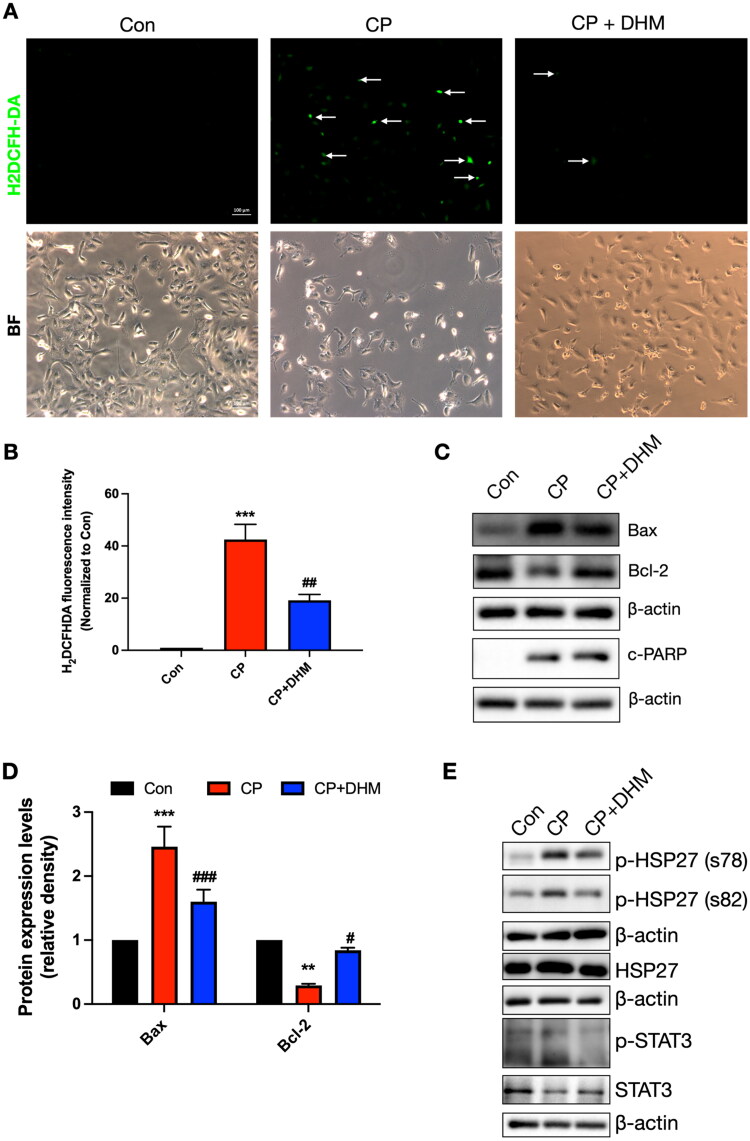

**Figure:**
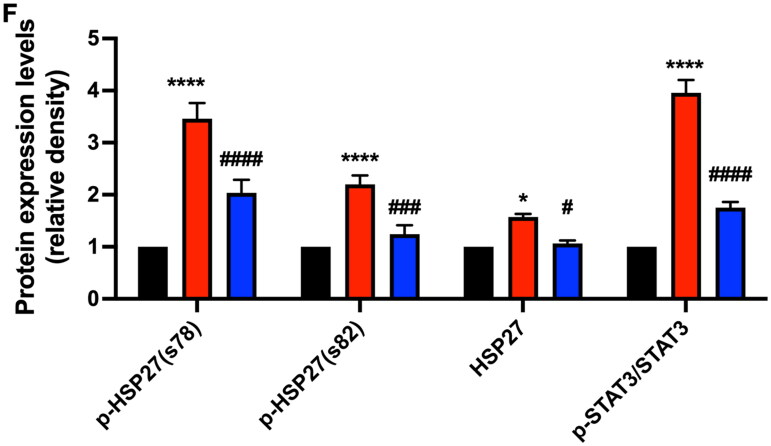

### 3.7. Gef protected against CP-induced cell damage in HK-2 cells

**Figure 7.**
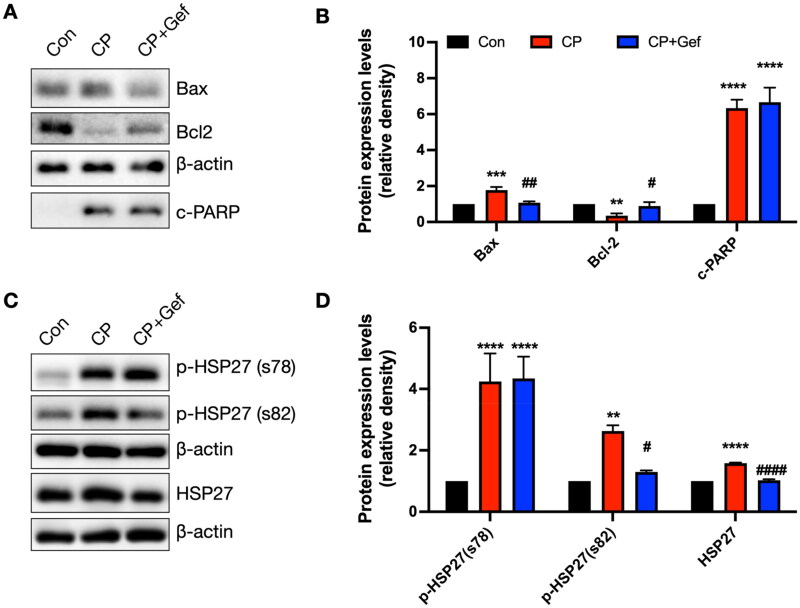
Effect of EGFR inhibitor gef on CP-induced HK-2 cell injury *in vitro*. (A) Immunoblotting images of bax, bcl-2, and c-PARP. (*n* = 3) (B) Evaluation of normalized bax, bcl-2, and c-PARP. (C) Immunoblotting images of HSP27 and p-HSP27 (s78, s82) in three categories. (D) Evaluation of normalized HSP27 and p-HSP27 (s78, s82). **, ***, ****p < .01, .001, .0001 versus sham category, respectively; #, ##, ####*p* < .05, .01, .0001 versus CP group, respectively.

To further verify that DHM inhibits renal injury by inactivating EGFR, HK-2 cells were pretreated with Gef before treatment with CP. The acquired Western blot data demonstrated that CP treatment upregulated the pro-apoptotic proteins such as Bax and c-PARP, and downregulated anti-apoptotic protein Bcl-2, whereas Gef prevented these changes, except c-PARP (Figure 7(A,B)). Moreover, Gef alleviated the activation/phosphorylation of HSP27 induced by CP (Figure 7(C,D)). The data indicated that EGFR inhibition significantly alleviated CP-induced HK-2 cell injury.

### 3.8. Si-HSP27 alleviated CP-induced cell damage in vitro in HK-2 cells

**Figure 8.**
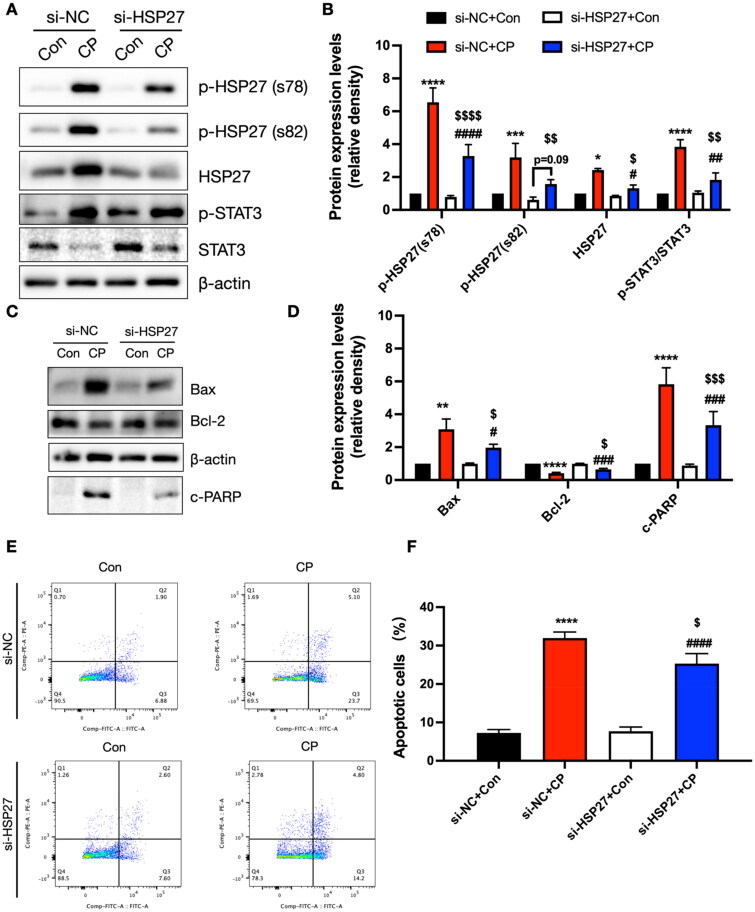
Impact of HSP27 inhibition on CP-induced cell injury *in vitro*. (A) Immunoblotting images of p-HSP27 (s78), p-HSP27 (s82), HSP27, p-STAT3, and STAT3 (*n* = 3). (B) Evaluation of normalized p-HSP27 (s78, s82), HSP27, p-STAT3, and STAT3. (C) Immunoblotting images of c-PARP, bax, and bcl-2 (*n* = 3). (D) Evaluation of normalized c-PARP, bax, and bcl-2. (E and F) Representative images of flow cytometry analysis (E) and evaluation of cell apoptosis (F) (*n* = 4). *, **, ***, ****p < .05, .01, .001 and .0001 *versus* si-NC + con category, respectively; #, ##, ###, ####*p* < .05, .01, .001 and .0001 *versus* si-HSP27 + con category, respectively; $, $$, $$$, $$$$ *p* < .05, .01, .001 and .0001 *versus* si-NC + CP category.

Previous data, including proteomics, immunoblotting, and immunofluorescence, demonstrated that HSP27 is critically involved in CP-induced AKI. To further validate this, si-HSP27 was utilized to detect the effect after blocking HSP27 in CP-induced cell injury. The data indicated that CP triggered the elevation of the levels of HSP27, p-HSP27, and p-STAT3, while si-HSP27 significantly prevented these changes (Figure 8(A,B)). Furthermore, CP induced mitochondrial-mediated apoptosis, manifested by the upregulation of Bax and c-PARP, along with the downregulation of Bcl-2. These changes were blocked by si-HSP27 (Figure 8(C,D)). Next, the role of si-HSP27 in CP-induced cell apoptosis was assessed using flow cytometry. Consistent with the results of immunoblotting data, the flow cytometry analysis data demonstrated that CP treatment increased the apoptosis of HK-2 cells, which was partially prevented by si-HSP27 (Figure 8(E,F)).

## 4. Discussion

The present study provides evidence that DHM can attenuate the increase of creatinine, BUN, KIM-1, NGAL, renal pathological damage, oxidative stress, mitochondrial damage, mitochondrial-mediated apoptosis, and epithelial cell injury in cisplatin (CP)-induced AKI, which may be involved in EGFR/HSP27/STAT3 pathway. CP is a commonly used chemotherapeutic drug to treat different solid tumors [62]. However, AKI is one of the common adverse effects of cisplatin that restrict its clinical application. CP is mainly reabsorbed in proximal tubular cells [63]. The renal tubular epithelial cell dysfunction/loss constitutes an important initiator of renal injury in AKI [64].

DHM, a flavonoid acquired from *Ampelopsis grossedentata*, exhibits various pharmacological properties. Several studies have reported that DHM exerts protective effects on various types of AKI [37,40,65]. Our previous research showed that DHM protected against cisplatin-induced AKI through mitigating oxidative/nitrative stress, inflammation ferroptosis [41]. Excessive generation of reactive oxygen species (ROS) was one of the earliest components in AKI. Increasing evidence indicates that the mitochondrion is the primary endogenous source of ROS, and ∼90% of ROS are generated in mitochondria [66]. Mitochondrial injury/dysfunction leads to decreased ATP synthesis, inflammation, mitochondrial ROS activation, and eventual cell death in renal epithelial cells [67,68]. Many studies have confirmed that mitochondrial dysfunction plays a key role in the pathogenesis of kidney diseases, such as diabetic kidney disease [69,70], ischemia/reperfusion-induced AKI [71–73] or CP-induced AKI [74–76]. Consistent with previous studies, our transmission electron microscopy results showed that mitochondrial lesions such as mitochondrial swelling, disruption of membrane integrity, and broken or absent cristae were observed in the kidneys of the CP-AKI mice, which were improved by DHM treatment. Moreover, our data indicated that DHM reduced cisplatin induced ROS activation in HK-2 cells *in vitro*.

Mitochondrial injury plays an important role in triggering apoptosis in response to various stress [77]. Bax and Bcl-2 are hall markers of mitochondria-mediated apoptosis involved in CP-AKI [78]. Our data showed that CP-induced Bax upregulation and Bcl-2 downregulation, which was inhibited by DHM treatment in mice. Consistent with *in vivo* results, *in vitro* data indicated that DHM significantly inhibited the upregulation of Bax and downregulation of Bcl-2 induced by cisplatin, thereby alleviating cisplatin induced HK-2 cell injury. Peroxisome proliferator-activated receptor γ coactivator-1α (PGC-1α), a key regulator of mitochondrial biogenesis and metabolic function, is predominantly distributed in the nucleus and activates transcription factors that transactivate the nuclear genes in renal tubular cells [79]. Studies observed that PGC-1α downregulation in the kidney plays an important role in the pathophysiological progression of various AKI [9,79–81]. Consistent with these research findings, our data also demonstrated that DHM not only alleviated mitochondrial morphological damage, but also significantly improved cisplatin induced the downregulation of PGC-1α. Novel strategies targeting the improvement of mitochondrial function could serve as promising therapeutic interventions in AKI.

To further study the molecular mechanism of DHM in improving cisplatin induced AKI, we conducted proteomic test in different experimental groups. Through proteomic data analysis, we found that HSP27 was significantly increased after cisplatin induced AKI, while DHM significantly reduced HSP27 activation. Several studies found that STAT3 is the downstream of HSP27[61,82]. Our data showed that DHM inhibited the phosphorylation of STAT3 induced by cisplatin. There have been some research reports on the important role of HSP27 in AKI [29,30,32]. To demonstrate the important role of HSP27 in cisplatin induced AKI, we blocked HSP27 using small interference techniques (si-HSP27), and the results showed that si-HSP27 significantly reduced cell apoptosis and activation of STAT3 induced by cisplatin *in vitro*. EGFR is an upstream of HSP27^19^. We blocked EGFR by using gefitinib and investigated its effect on cisplatin induced cell damage. Our results showed that blocking EGFR significantly reduced cisplatin induced cell apoptosis and phosphorylation/activation of HSP27 *in vitro.* Next, we examined the effects of DHM treatment on EGFR/HSP27/STAT3 signaling pathway *in vivo*. Our results revealed that DHM remarkably inhibited the activation/phosphorylation of EGFR, HSP27, and STAT3 induced by cisplatin. All the data indicated that HSP27 could be an important therapeutic target for CP-induced AKI. Extended investigation is warranted to elaborate on the critical role of HSP27 and its associated mechanisms in CP-based chemotherapy.

## 5. Conclusion

**Figure 9.**
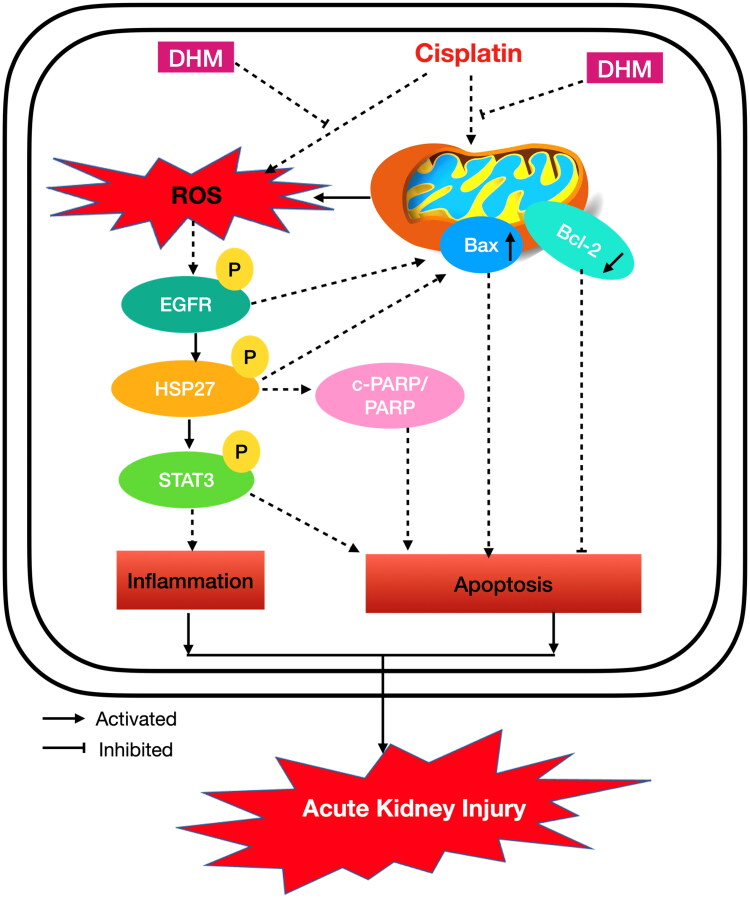
Schematic representation of the protective function of DHM on renal oxidative stress, inflammation, and mitochondrial-induced apoptosis by regulating the EGFR/HSP27/STAT3 signaling pathway in CP-induced AKI. DHM: dihydromyricetin; EGFR: epidermal growth factor receptor; HSP27: heat shock protein 27; ROS: reactive oxygen species; STAT3: signal transducer and activator of transcription 3.

In summary, our study highlights that DHM protects against renal injury by improving inflammation, oxidative stress, and mitochondrial-mediated apoptosis, potentially by targeting the EGFR/HSP27/STAT3 signaling pathway (Figure 9). Our data provide robust evidence that HSP27 might as a promising target for the treatment of AKI and DHM is a potential therapeutic drug for reducing CP-induced AKI in patients undergoing cancer therapy.

## Supplementary Material

Supplementary_Data_clean.docx

## Data Availability

All data generated or analyzed in this study are included in this article. Further inquiries are available from the corresponding author on reasonable request.
