# Supplementary material for: Dihydromyricetin protects against cisplatin-induced renal injury and mitochondria-mediated apoptosis via the EGFR/HSP27/STAT3 signaling pathway
Source: Ren Fail. 2025 Apr 14;47(1):2490202. doi: 10.1080/0886022X.2025.2490202 (PMC12001862; doi:10.1080/0886022X.2025.2490202)
Supplement: Supplementary_Data_clean.docx [file IRNF_A_2490202_SM8375.docx]

﻿**Supplemental materials**

**Dihydromyricetin protects against cisplatin-induced renal injury and mitochondria-mediated apoptosis via the EGFR/HSP27/STAT3 signaling pathway**

Zheming Xu^1, 2, #^, Minjing Zhang^1, 2, #^, Xue Zhang^1, 2, #^, Huirong Han^3^, Weifeng Ye^2^, Zhenjie Chen^2^, Zhisu Lv^2^, Yang Liu^2^, Zhengye Liu^4^, Jianguang Gong^5^, Bin Zhu^5^, Suhan Zhou^6^, Runzhi Zhu^2^, Chang Tao^1, 2^, Jianhua Mao^2^, Gensheng Zhang^1, 2*^, Xiang Yan^1, 2*^

﻿

﻿**SUPPLEMENTAL FIGURES AND LEGENDS**

Figure S1. Effect of DHM on TNF-α and IL-1β protein levels after CP-induced AKI *in vivo.* (A-B) Immunofluorescence images of TNF-α (A), ﻿with quantitation depicted in (B) (n=3/group). (C-D) Immunofluorescence images of IL-1β (C), ﻿with quantitation depicted in (D) (n=3/group). ** *P* < 0.01 *versus* sham group. #*P* < 0.05 *versus* CP group.

**
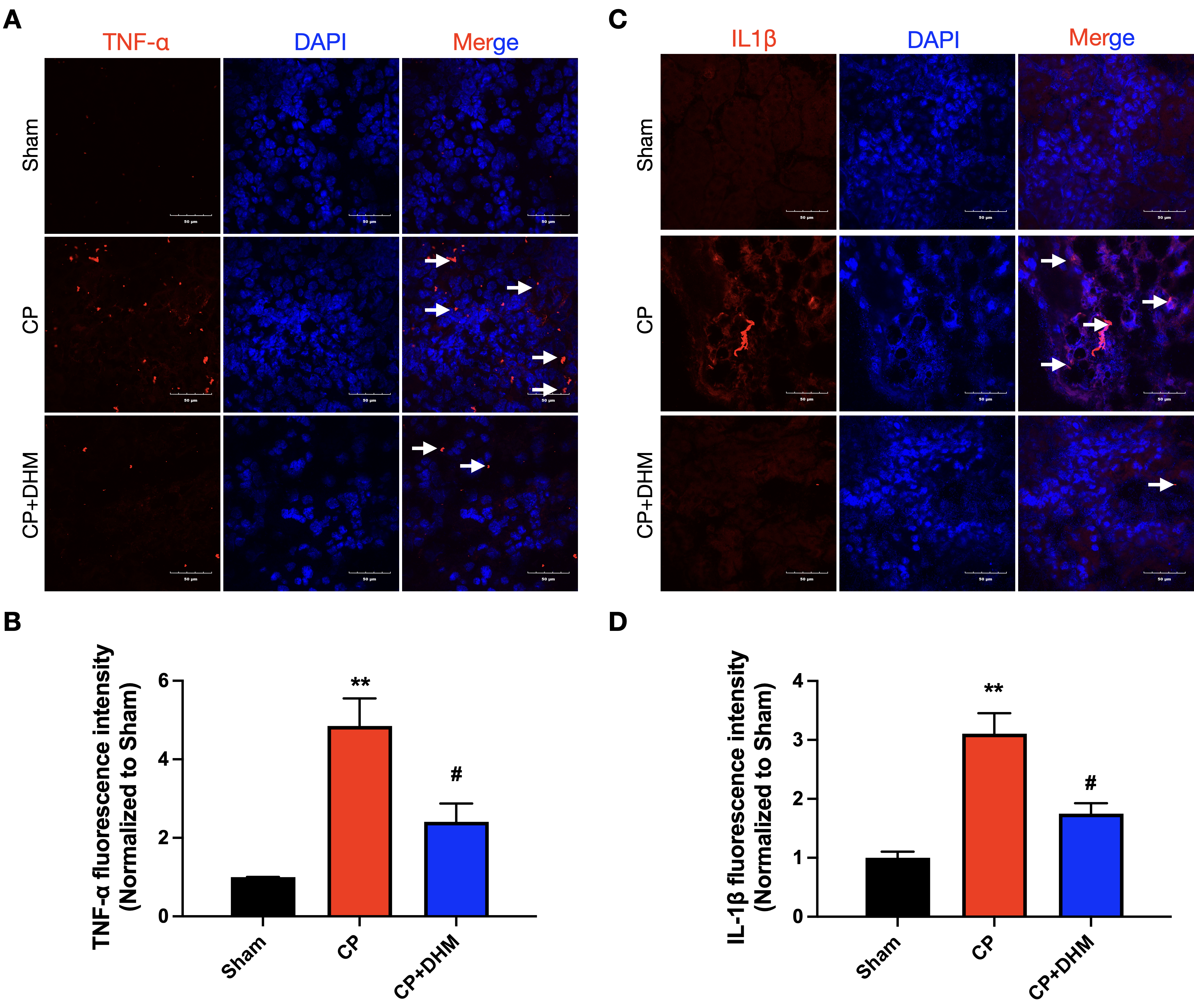
**

Figure S2. TMT-quantitative proteomic data in Sham, CP, and CP+DHM groups. (A) Volcano plot of ﻿DEPs in the three categories. Blue points downregulated proteins; red points, upregulated proteins; gray points, proteins with no statistically significant differences. (B) KEGG analysis of DEPs. (C) GO analysis of DEPs. (D) Heatmap representation of DEPs between CP and sham categories.

**

**

**

**

Figure S3. Effect of DHM on IL-1Ra and BPIFA2 protein levels after CP-induced AKI. (A-B) Immunofluorescence confocal images of IL-1Ra (A), ﻿with quantitation depicted in (B) (n=3/group). (C-D) Immunofluorescence confocal images of BPIFA2 (C), ﻿with quantitation depicted in (D) (n=3/group). ***, **** *P* < 0.001 and 0.0001 *versus* sham group. ####*P* < 0.0001 *versus* CP group.

**
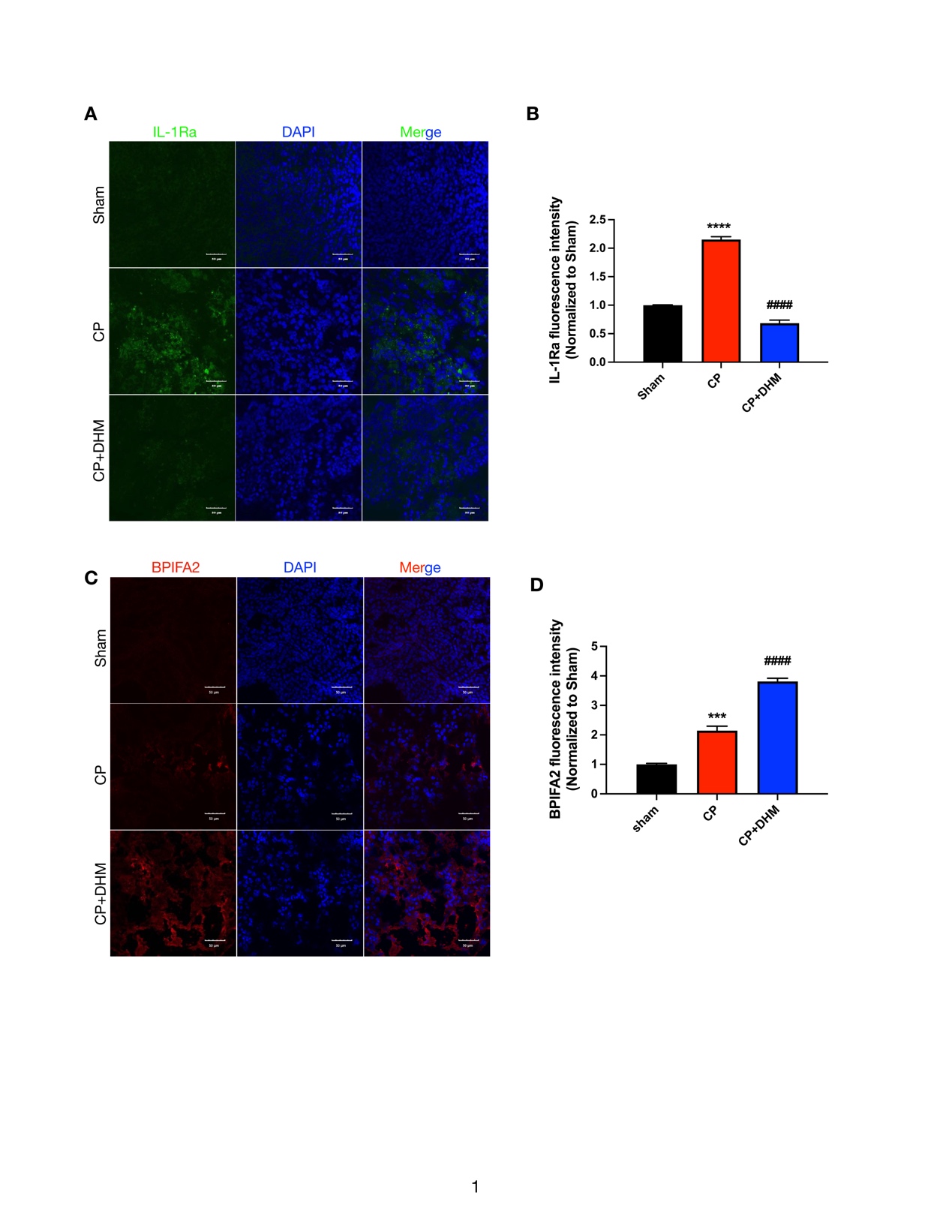
**
